# Enhancing Interferon Regulatory Factor 7 Mediated Antiviral Responses and Decreasing Nuclear Factor Kappa B Expression Limit HIV-1 Replication in Cervical Tissues

**DOI:** 10.1371/journal.pone.0131919

**Published:** 2015-06-29

**Authors:** Christiane Rollenhage, Sherrill L. Macura, Melissa J. Lathrop, Todd A. Mackenzie, Gustavo F. Doncel, Susana N. Asin

**Affiliations:** 1 Research Service, V. A. Medical Center, White River Junction, VT, United States of America; 2 Department of Microbiology and Immunology, Geisel School of Medicine at Dartmouth, Lebanon, NH, Unites States of America; 3 Department of Medicine, Geisel School of Medicine at Dartmouth, Lebanon, NH, United States of America; 4 Department of Community and Family Medicine, Geisel School of Medicine at Dartmouth, Lebanon, NH, United States of America; 5 CONRAD, Eastern Virginia Medical School, Norfolk, VA, United States of America; Temple University School of Medicine, UNITED STATES

## Abstract

Establishment of a productive HIV-1 infection in the female reproductive tract likely depends on the balance between anti-viral and pro-inflammatory responses leading to activation and proliferation of HIV target cells. Immune modulators that boost anti-viral and depress pro-inflammatory immune responses may decrease HIV-1 infection or replication. Polyinosinic:polycytidylic [Poly (I:C)] has been reported to down-regulate HIV-1 replication in immune cell subsets and lymphoid tissues, yet the scope and mechanisms of poly (I:C) regulation of HIV-1 replication in the cervicovaginal mucosa, the main portal of viral entry in women remain unknown. Using a relevant, underexplored *ex vivo* cervical tissue model, we demonstrated that poly (I:C) enhanced Interferon Regulatory Factor (IRF)7 mediated antiviral responses and decreased tissue Nuclear Factor Kappa B (NFκB) RNA expression. This pattern of cellular transcription factor expression correlated with decreased HIV-1 transcription and viral release. Reducing IRF7 expression up-regulated HIV-1 and NFκB transcription, providing proof of concept for the critical involvement of IRF7 in cervical tissues. By combining poly (I:C) with a suboptimal concentration of tenofovir, the leading anti-HIV prophylactic microbicide candidate, we demonstrated an earlier and greater decrease in HIV replication in poly (I:C)/tenofovir treated tissues compared with tissues treated with tenofovir alone, indicating overall improved efficacy. Poly (I:C) decreases HIV-1 replication by stimulating IRF7 mediated antiviral responses while reducing NFκB expression. Early during the infection, poly (I:C) improved the anti-HIV-1 activity of suboptimal concentrations of tenofovir likely to be present during periods of poor adherence i.e. inconsistent or inadequate drug use. Understanding interactions between anti-viral and pro-inflammatory immune responses in the genital mucosa will provide crucial insights for the identification of targets that can be harnessed to develop preventative combination strategies to improve the efficacy of topical or systemic antiviral prophylactic agents and protect women from HIV-1 and other sexually transmitted infections.

## Introduction

Poly (I:C) is a ligand for several pathogen recognition receptors including toll like receptor 3 (TLR)3, retinoic acid-inducible gene 1 (RIG-1) and melanoma differentiation-associated gene 5 (MDA5), which modulate inflammatory and anti-viral responses [1–3]. While activation of the anti-viral transcription factor interferon regulatory factor (IRF) 7, is specific to the RIG-1/MDA5 pathway [4,5], IRF3 and the nuclear factor κB (NFκB) family member RelA are activated by both TLR3 and RIG-1/MDA5 signaling pathways [4,6,7]. Poly (I:C) up-regulates RIG-1 and MDA5 expression following a 24 hour (hr) treatment of genital epithelial cells [8]. Up-regulation of receptor expression was associated with increased Interleukin (IL)-8, IL-6 and IL-1β expression [8]. Poly (I:C) has also been reported to enhance expression of the anti-viral factors trappin-2/elafin and macrophage inflammatory protein 3 alpha in genital epithelial cells [9,10].

Anti-HIV-1 activity of poly (I:C) has been reported in peripheral blood mononuclear cells (PBMCs), subsets of CD4^+^ immune cells and human lymphoid tissues [11–14]. Poly (I:C) decreased HIV-1 replication in immature dendritic cells (imDC) and monocyte-derived macrophages (MDM), by up-regulating expression of type I Interferon (IFN) inducible anti-viral factors such as APOBEC3G and tetherin [11,12]. Poly (I:C) treated MDM also had increased levels of CC chemokines, some being the ligands for the HIV-1 co-receptor CCR5, which may have inhibited HIV-1 binding and internalization [11].

The scope and mechanism of poly (I:C) modulating HIV-1 replication in female reproductive tract tissues, mucosal sites of HIV-1 exposure that display complex interactions between epithelial and immune cells, remain unknown. Since poly (I:C) is an immune modulator with reported anti-HIV-1 activity, we evaluated its potential alone and in combination with the antiretroviral tenofovir (TFV), a leading microbicide candidate, at suboptimal concentrations to regulate HIV-1 replication. In this study, poly (I:C) decreased HIV-1 replication in *ex vivo* cervical tissues by increasing IRF7 mediated antiviral responses and decreasing RelA RNA expression. In turn, this pattern of transcription factors and Type I Interferon (IFN) expression was associated with reduced levels of HIV-1 transcription. Down-regulation of IRF7 expression by IRF7 specific siRNA enhanced HIV-1 and RelA transcription, underscoring the role of IRF7 in decreasing HIV-1 replication in cervical tissues. By using poly (I:C) in combination with a suboptimal concentration of TFV likely to be present under inconsistent use of the microbicide, we demonstrated an earlier and greater decrease in HIV-1 replication compared with tissues treated with TFV alone, suggesting synergistic effect and improved microbicide efficacy.

## Materials and Methods

### Tissue samples

Cervical tissues were procured from HIV-1 sero-negative women who were undergoing hysterectomy at Dartmouth-Hitchcock Medical Center (DHMC) for benign medical conditions including prolapse and uterine fibroids. All tissues were obtained according to a protocol approved by the Committee for the Protection of Human Subjects at Dartmouth College (CPHS# 23483). Participants written informed consent was obtained prior to conducting experiments in cervical tissues.

Non-polarized cervical tissues were established in 48-well plates as described [15–18]. Using our culture conditions, tissue explants can be maintained for up to 21 days without significant decrease in viability, as determined by LDH viability assay (Cytotoxicity Detection Kit, Roche, Indianapolis, IN).

### HIV-1 infection

HIV-1 stocks were generated in human PBMCs. Tissues were infected with 10^4^ cell-free R5-tropic HIV-1_BaL_, the 50% Tissue Culture Infectious Dose (TCID_50_)/ml. After overnight incubation at 37°C, tissues were washed to remove residual input virus, and cultured for up to 21 days in Leibowitz (L) 15 medium supplemented with 10% heat inactivated fetal bovine serum (Hyclone, Logan, UT), 2 mM glutamine (GIBCO, Grand Island, NY), 50 unit/ml penicillin and 50 μg/ml streptomycin (complete L15, GIBCO, Grand Island, NY). A sample of the culture supernatants was collected after the final wash (day 0), and on days 4, 7, 11, 14, 18 and 21 after infection. On each day, one half of the culture supernatant was removed, and replenished with an equivalent volume of fresh media. Day’s 0, 11 and 21 supernatants were evaluated for HIV-1 p24 antigen by ELISA (Perkin Elmer, Boston, MA).

### PBMCs isolation and infection

PBMCs were isolated by Ficoll-Paque Plus (Amersham, Picataway, NJ) as described [19] and incubated over night at 37°C in complete phenol red-free RPMI-1640 (GIBCO, Grand Island, NY). Next day, cells were resuspended at 2x10^6^ cells/ml, and infected with 50 TCID_50_/ml of R5-tropic HIV-1_BaL_. After over night infection at 37°C, PBMCs were washed, resuspended in medium and cultured for 5 days.

### Poly (I:C) and TFV treatment

Tissues or PBMC were treated with poly (I:C) (InvivoGen, San Diego, CA) at 20 μg/ml and/or TFV at 10 μg/ml for 6 hrs before HIV-1 infection. Poly (I:C) was added to designated wells after washing the residual input virus and replenished every three days. Tenofovir drug substance was kindly provided by CONRAD (Arlington, VA).

### Nucleic acid isolation

Genomic DNA was isolated on days 11 and 21 using the QIAmp DNA mini kit (Qiagen, Valencia, CA). When indicated, RNA was isolated from cervical tissues or PBMCs before and on days one, three or five after infection. Tissues were homogenized with Omni tissue homogenizer (Omni, Kennesaw, GA). Tissue lysate supernatants were subjected to RNA isolation using the RNeasy-Plus kit (Qiagen, Valencia, CA). Cells were lysed in RLT-plus lysis buffer without previous homogenization. Five μg of total tissue RNA or one μg of total cellular RNA were reverse transcribed using Superscript III reverse transcriptase (Invitrogen, Carisbad, CA). The resulting cDNA was evaluated for gene expression by real-time PCR using SYBR-green® (Applied Biosystems, Warrington, UK).

### HIV-1 reverse transcription

HIV-1 DNA was detected by a two-step quantitative real-time PCR amplification. Genomic DNA (250 ng) was amplified using primers specific for the HIV-1 Gag protein. The first round amplification was performed for 15 cycles using GAG1 and SK431 sense and antisense primers. Five μl of this PCR product were amplified in a second round reaction using GAG1 and antisense GAG2 primers. HIV-1 DNA amplifications were normalized to human β-actin [20].

### HIV-1 integration

HIV-1 integrated DNA was detected by a two-step-real-time PCR assay. The first-round amplifies the DNA sequence between the HIV-1 sequence and the nearest chromosomal ALU elements whereas the nested PCR amplifies HIV-1 PCR products pre-amplified in the first round. The first-round amplification was performed for 12 cycles using primers targeting ALU elements (Alu1.24 and Alu2.25) and an HIV-1 specific primer extended at its 5' end with a lambda phage-specific heel sequence (L-M667.42). Five μl of this PCR product were used in a second round amplification with LambdaT.19 and M661.L24 primers targeting the heel-specific sequence and the HIV-1 *gag* gene [21,22]. Results were normalized to human β-actin.

### Gene transcription

HIV-1 RNA was detected with primers complementary to the flanking sequence of the common splice donor and acceptor sites of the *tat* and *rev* genes. All transcription values were normalized to endogenous human GAPDH. Primer sequences to detect HIV-1 reverse transcription; viral integration and transcription as well as receptor, type I IFN and transcription factor expression are described in Table 1.

**Table 1:** Primer sequences to detect reverse transcribed and integrated HIV-1 DNA, as well as HIV-1 and cellular receptor, transcription factor and house keeping gene expression.

Sequence	Gene	Size
GAG1: 5’-TCAGCCCAGAAGTAATAC-3’	Gag	220
SK431: 5’-TGCTATGTCAGTTCCCCT-3’		
GAG2: 5’-CACTGTGTTTAGCATGGTGTTT-3’	Gag	81
ALU1.24: 5’-TCCCAGCTACTGGGGAGGCTGAGG-3’	Alu/Gag	NA
ALU2.25: 5’-GCCTCCCAAAGTGCTGGGATTACAG -3’		
L-M667.42:5’ATGCCACGTAAGCGAAACTCTGGCTAACTAGGGAACCCACTG-3’		
LambdaT.19: 5’-ATGCCACGTAAGCGAAACT-3’	Gag	158
M661.L24: 5’-CCTGCGTCGAGAGAGCTCCTCTGG-3’		
Sense: 5’-CACTCTTCCAGCCTTCCTTCC-3’	β-actin	331
Antisense: 5’-CTGTGTTGGCGTACAGGTCT-3’		
Sense: 5’-CAGGAAGAAGCGGAGACAGC-3’	Tat/Rev	175
Antisense: 5’-CACTAATGGACCGGATC-3’		
Sense: 5’-CAGACCTCCTCTTGGCTTCG-3’	RIG-1	207
Antisense: 5’-GCTATCCAGGGAAGACACACC-3’		
Sense: 5’-GCACGGCTCTGGAAACAC-3’	TLR3	68
Antisense: 5’-GTGGACGTGAGACAGACCCTTT-3’		
Sense: 5’-CCCCACGCTATACCATCTACC-3’	IRF7	152
Antisense: 5’-GCTATCCAGGGAAGACACACC-3’		
Sense: 5’-GCTATGCCCTCTGGTTCTGTGTGG-3’	IRF3	177
Antisense: 5’-GGGTGGCTGTTGGAAATGTG-3’		
Sense: 5’-CCTGTCCTTTCTCAT CCCA-3’	RelA	83
Antisense: 5’-AGCTGCCAGAGTTTCGGTT-3’		
Sense: 5’-TCCCATCACCATCTTCCAG-3’	GAPDH	77
Antisense: 5’-GACTCCACGACGTACTCA-3’		
Sense: 5’-CTC TAC CAG CAA CTG AAT GAC-3’	IFNα	175
Antisense: 5’CTG CTC TG ACA ACC TCC C-3’		
Sense: 5’-GGC ACA ACA GGT AGT AGG CGA-3’	IFNβ	175
Antisense: 5’-GTA GTG GAG AAG CAC AAC AGG AG-3’		

### IRF7 silencing experiments

IRF7 targeting (sc-38011) and random [r, (sc-37007)] siRNAs were purchased from Santa Cruz (Dallas, TX). Nanoparticles encapsulating IRF7 targeting and rsiRNA were generated following the manufacturer’s instructions (Genelantis, San Diego, CA). Briefly, 2μl of transfection reagent in 50μl serum free media were pre-incubated with 50μl of 40nM siRNA for 15 minutes at room temperature. This mix of nanoparticles encapsulating siRNA was diluted to 50% with 2x media and 200 μl were added to five pieces of cervical tissues in individual wells of a 48-well plate. Following a 24 hr incubation, culture supernatants were diluted 1-fold with media and incubated for additional 24 hrs. Cervical tissues were washed once, and infected with R5-tropic HIV-1_BaL_. Following overnight incubation at 37°C, tissues were washed and fresh culture media was added back to the tissues. Total tissue RNA was isolated before and on days 1 and 3 after infection and evaluated for HIV-1, IRF7, RelA and IFNα transcription.

### Fluorescence Activated Cell Sorting (FACS) experiments

Ectocervical tissues (8–10 pieces) were digested with collagenase D (Roche Diagnostics, Indianapolis, IN) at 5mg/ml in complete L15 for not more that 1 and a half hrs at 37°C. Single cell suspensions were suspended in flow buffer (1X PBS supplemented with 2% FBS and 0.05% sodium azide) and fixed in flow buffer containing 0.5% paraformaldehyde. This standardize cell isolation procedure ensures maximum cell yield with minimal effects or surface antigen expression and cell viability [23]. Cells were stained with a combination of PE-conjugated anti-CD3 (clone HIT3a) and APC-conjugated anti-CD8 (clone Sk1) monoclonal antibodies. All antibodies were purchased from BioLegend (San Diego, CA). Data were acquired on a BD FCASCanto II (BD Biosciences) using FACSDiva software (BD Biosciences) and analyzed with FloJo version 10.0.6 (Ashland, OR). CD4^+^ T cells were defined as CD3^+^ and CD8^-^.

### Statistical analysis

Our experimental design fits a hierarchical model to account for repeated measurements, each triplicated, within tissues. In particular, we included a random intercept for tissue and triplicate within tissue, in addition to fixed effects for the day and treatment. Analysis of datasets for HIV-1 reverse transcription, integration and p24 containing two groups (untreated and poly (I:C)-treated tissues) at days 11 and 21 after infection was performed by paired Student’s t-test using triplicates from each individual donor after logarithmic transformation to achieve normality. Data from transcription experiments were expressed as arithmetic means and compared by paired Student’s t-test. P values of <0.05 were considered significant.

## Results

### Poly (I:C) decreases HIV-1 replication in cervical tissues

**Fig 1:**
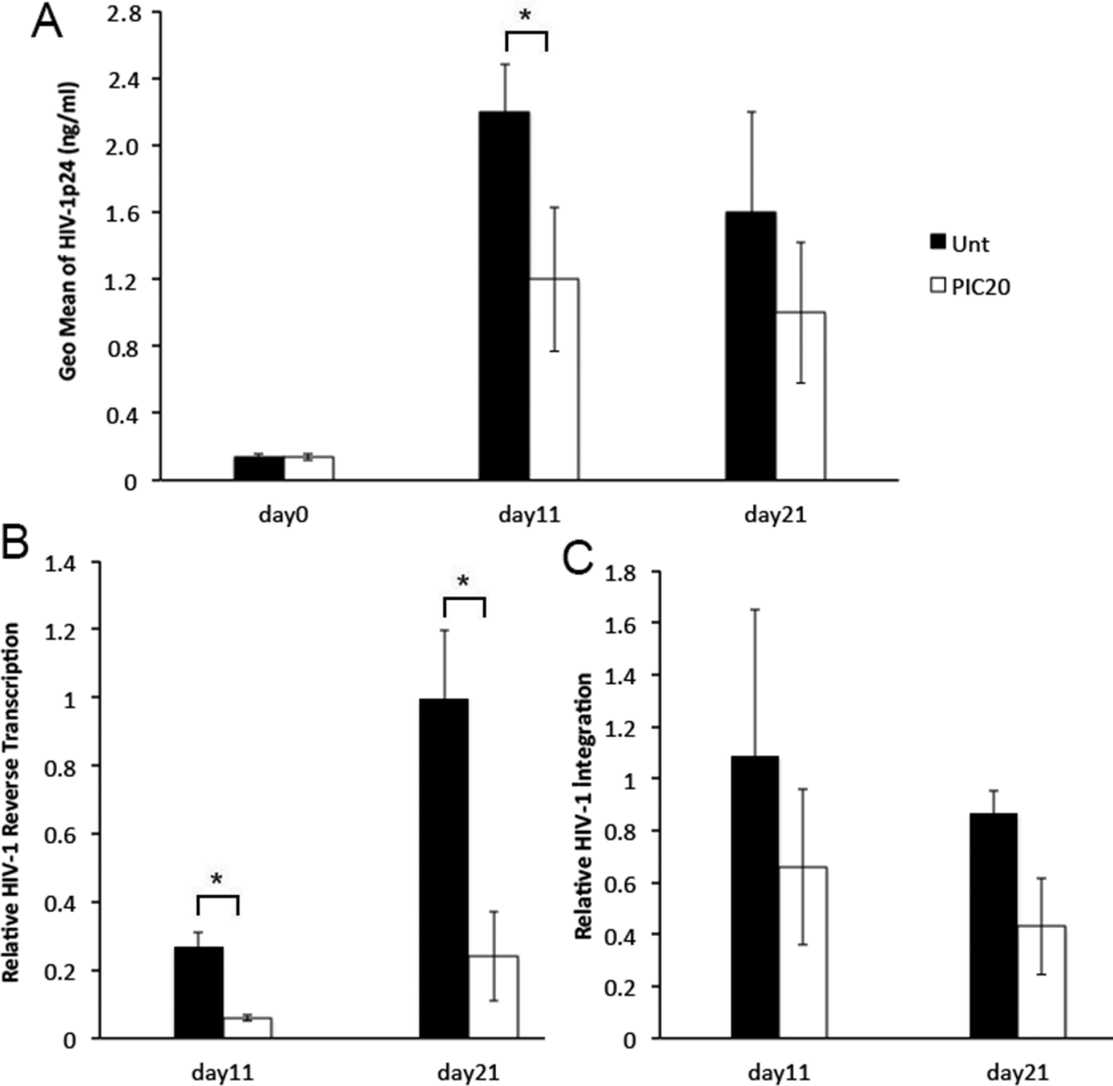
Poly (I:C) decreases HIV-1 replication in cervical tissues. (A) HIV-1 p24 levels (ng/ml) in HIV-1 infected cervical tissues left untreated or treated with poly (I:C) at 20 μg/ml were measured after washing the residual input virus (day 0), and again on days 11 and 21 after infection. Results are shown as the geometric mean ± STDEV from triplicate values of 12 individual donors. *p = 0.00005 and p = 0.07 between untreated and poly (I:C) treated tissues at days 11 and 21 after infection, respectively. (B) Levels of HIV-1 reverse transcription and (C) integration in donor matched HIV-1 infected cervical tissues left untreated or treated with poly (I:C) at 20 μg/ml were quantified by RT-PCR on days 11 and 21 after infection. All data was normalized to human β actin. For HIV-1 reverse transcription and integration, day 11 values in untreated control tissues were set to 1. Day 11 values in poly (I:C) treated tissues or days 21 values in untreated or poly (I:C) treated tissues were normalized to 1. Results are shown as the relative geometric mean ± STDEV from triplicate values of 8 and 12 individual donors on days 11 and day 21 respectively. *p = 0.004 and p = 0.0009 between untreated and poly (I:C) treated tissues at days 11 and 21 after infection, respectively.

Poly (I:C) inhibits HIV-1 replication in PBMCs, imDC, MDM and human lymphoid tissues [11–14]. To test whether poly (I:C) decreased HIV-1 replication at mucosal sites of HIV-1 exposure, we conducted infectivity experiments where cervical tissues were established as described [24] and left untreated or treated with poly (I:C) prior to infection with R5 tropic HIV-1_BaL_. After overnight incubation, tissues were washed to remove residual input virus (Day 0) and cultured for 21 days. On day 0, poly (I:C) was added back to designated tissues and replenished every three days. Tissue culture supernatants were evaluated for HIV-1 p24 levels on days 11 and 21 after infection (Fig 1A). At these time points, tissue genomic DNA was extracted and assessed for HIV-1 reverse transcription (Fig 1B) and viral integration (Fig 1C). Day 11 is one of the earliest time points where we consistently detect HIV-1 DNA expression. In general, P24 release peaks at day 11. Day 21 is the day where we terminate the experiment [25]. Results from these experiments revealed a decrease in HIV-1 replication in poly (I:C) treated compared with untreated control tissues at day 11 after infection (p = 0.00005), which was not sustained through day 21 (p = 0.07) (Fig 1A). Decreased HIV-1 replication was not associated with a poly (I:C) mediated decrease in tissue viability as evaluated by lactate dehydrogenase (LDH) levels in tissue culture supernatants (data not shown). HIV-1 reverse transcription was significantly reduced by poly (I:C) at both days 11 (p = 0.004) and 21 (p = 0.0009) after infection (Fig 1B). Assuming one copy of viral DNA is integrated per infected cell, and using HIV-1 integration as a surrogate of number of HIV-infected cells [26], we detected a modest not statistically significant reduction in number of cells with integrated HIV-1 in poly (I:C) treated compared with untreated control tissues at days 11 (p = 0.25) and 21 (p = 0.36) (Fig 1C).

### Poly (I:C) decreases HIV-1 transcription in cervical tissues by enhancing IRF7 mediated antiviral responses and decreasing RelA expression

**Fig 2:**
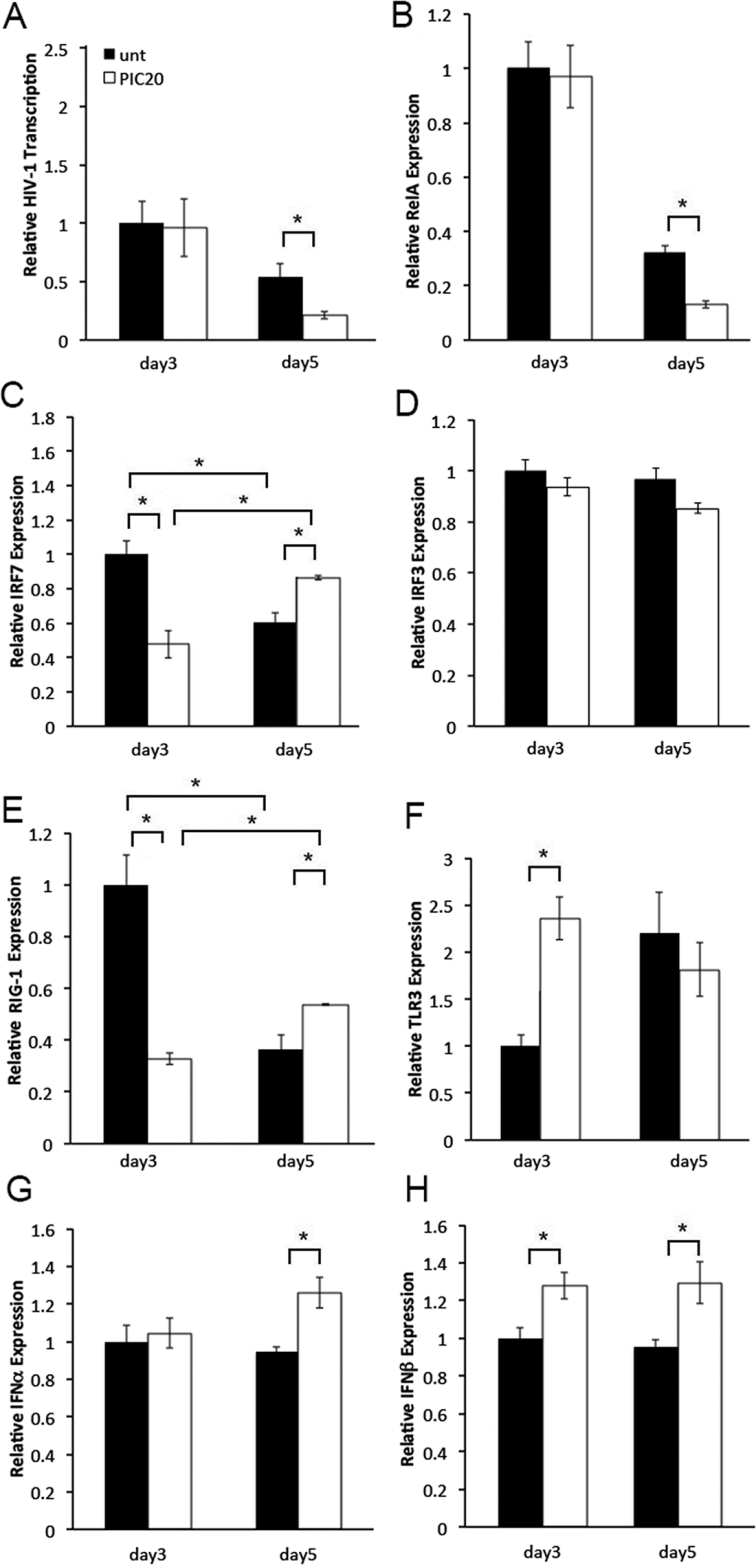
Poly (I:C) decreases HIV-1 transcription in cervical tissues by enhancing IRF7 mediated antiviral responses and reducing RelA expression. (A) Levels of HIV-1, (B) RelA, (C) IRF7, (D) IRF3, (E) RIG-1, (F) TLR3, (G) IFNα and (H) IFNβ transcription in HIV-1-infected cervical tissues left untreated or treated with poly (I:C) at 20 μg/ml were quantified by RT-PCR on days 3 and 5 after infection. All data was normalized to glyceraldehyde 3-phophate (GAPDH). For each gene, day 3 values in untreated control tissues were set to 1. Day 5 values in untreated control tissues or days 3 and 5 values in poly (I:C) treated tissues were normalized to 1. Results were consistent among three donors and are shown as the mean ± STDEV from one representative experiment with each condition tested in triplicate. * p<0.05 for untreated and poly (I:C) treated tissues.

Poly (I:C) induces anti-viral and pro-inflammatory responses [1,3]. Given the moderate effect on number of HIV infected cells, poly (I:C) may have decreased HIV-1 replication by modulating post-integration events, potentially viral transcription. To test this hypothesis, we compared expression levels of the HIV-1 early transcripts Tat and Rev between poly (I:C) treated and untreated control tissues on days 3 and 5 after infection. We selected these early time points because we wanted to evaluate gene transcription following immune cell activation yet before immune cell depletion by HIV-1 [25]. On day 3, we detected similar levels of HIV-1 RNA expression in poly (I:C) treated and untreated control tissues. Conversely, on day 5, HIV-1 transcription was significantly down regulated by poly (I:C) (Fig 2A). To address whether poly (I:C) down-regulated HIV-1 transcription by modulating anti-viral and pro-inflammatory responses, we compared expression levels of the anti-viral IRF3 and IRF7, and pro-inflammatory RelA transcription factors between poly (I:C) treated and untreated control tissues from the same experiment. On day 3, IRF7 expression was down regulated in poly (I:C) treated compared with untreated control tissues. Conversely, on day 5 we detected enhanced IRF7 expression in poly (I:C) treated than in untreated control tissues (Fig 2C). Increased IRF7 expression on day 5 correlated with enhanced IFNα expression and decreased HIV-1 transcription in poly (I:C) treated compared with untreated control tissues (Fig 2A, 2C and 2G). Decreased IRF7 expression on day 3 was associated with similar levels of either IFNα and HIV-1 transcription in poly (I:C) treated and untreated control tissues. IRF3 expression levels were not impacted by poly (I:C) throughout day 5 (Fig 2D). Unlike day 3 when no changes were observed, on day 5, RelA transcription levels were decreased by poly (I:C) (Fig 2B). Down-regulation of RelA expression correlated with decreased HIV-1 transcription (Fig 2A and 2B).

Poly (I:C) activates TLR3 and RIG-1/MDA5 signaling pathways [1,3,4]. To determine whether modulation of IRF7 and RelA transcription was associated with changes in receptor expression triggered by poly (I:C), we compared transcription levels of RIG-1 and TLR3 receptors between poly (I:C) treated and untreated control tissues from the same experiment. Kinetics of RIG-1 expression reflected those of its specific downstream targets IRF7 (Fig 2C and 2E). Enhanced RIG-1 expression correlated with decreased RelA transcription on day 5 (Fig 2E and 2B)

Kinetics of TLR3 expression was not associated with those of its specific downstream targets RelA or IRF3 through day 5 after infection (Fig 2B, 2D and 2F). Enhanced TLR3 expression on day 3 was associated with up-regulation of IFNβ expression (Fig 2F and 2H).

### Poly (I:C) increases IRF7 and RelA expression in PBMCs

**Fig 3:**
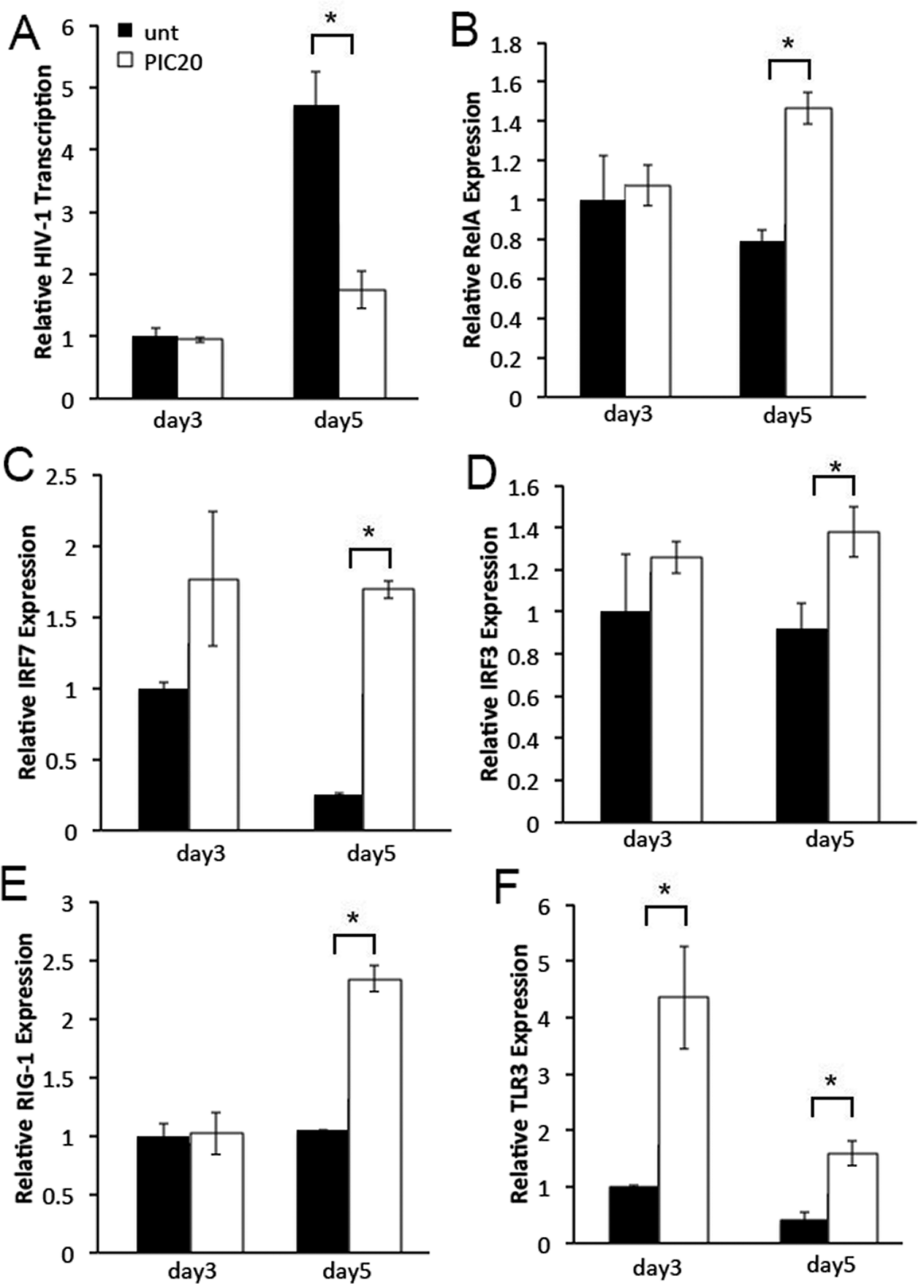
Poly (I:C) decreases HIV-1 transcription by increasing IRF7 expression in PBMCs. (A) Levels of HIV-1, (B) RelA, (C) IRF7, (D) IRF3, (E) RIG-1, and (F) TLR3 transcription in HIV-1 infected PBMCs left untreated or treated with poly (I:C) at 20 μg/ml were quantified by RT-PCR on days 3 and 5 after infection. All data was normalized to GAPDH. For each gene, day 3 values in untreated control tissues were set to 1. Day 5 values in untreated control tissues or days 3 and 5 values in poly (I:C) treated tissues were normalized to 1. Results were consistent among three donors and are shown as the mean ± STDEV from one representative experiments with each condition tested in triplicate. * p<0.05 for untreated and poly (I:C) treated cells.

To evaluate whether immune cells reflected mechanisms of poly (I:C) regulation of HIV-1 replication in cervical tissues, we conducted poly (I:C) experiments using non-stimulated PBMCs as a surrogate of sub-mucosal leukocytes. In these experiments, PBMCs were treated with poly (I:C) prior to overnight infection with HIV-1. The next day, cells were washed to remove residual input virus. Poly (I:C) was added back to designated wells and kept in cultures through day 5. Total cellular RNA was isolated on days 3 and 5 after infection, and evaluated for HIV-1, cellular transcription factor and receptor RNA expression. On day 3, poly (I:C) had no effect on HIV-1, IRF7, RelA or IRF3 RNA expression compared with that in untreated control cells (Fig 3A, 3B, 3C and 3D). Conversely on day 5, levels of HIV-1 transcription were decreased in poly (I:C) treated compared with untreated control PBMCs (Fig 3A). Decreased HIV-1 transcription on day 5 correlated with enhanced IRF7, RelA and IRF3 RNA expression (Fig 3B, 3C and 3D). Kinetics of cellular transcription factors expression was associated with either no change, or enhanced RIG-1 transcription levels on days 3 and 5 after infection, respectively (Fig 3E). In contrast, TLR3 expression was enhanced throughout day 5 (Fig 3F).

**Fig 4:**
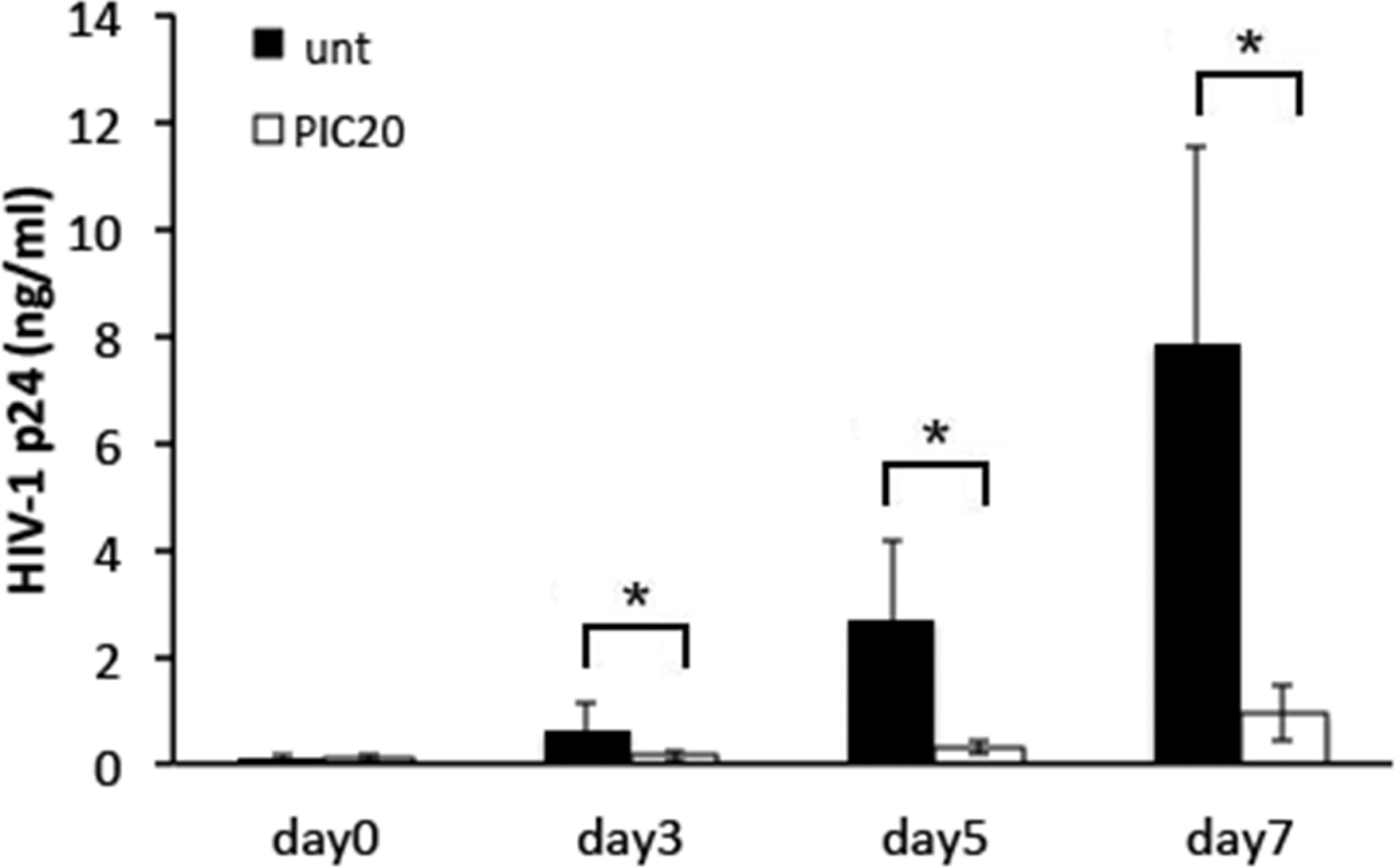
Poly (I:C) decreases HIV-1 replication in PBMCs. HIV-1 p24 levels (ng/ml) in HIV-1 infected PBMCs left untreated or treated with poly (I:C) at 20 μg/ml were measured after washing the residual input virus (day 0), and again on days 3, 5 and 7 after infection. Results are shown as the mean ± STDEV from three experiments with each condition tested in triplicate. * p = 0.008, p = 0.01 and p = 0.009 for days 3, 5 and 7 after infection between untreated and poly (I:C) treated PBMCs.

As expected, decreased HIV-1 gene expression correlated with lower HIV-1 p24 levels in poly (I:C) treated compared with untreated control cells (Fig 4).

### Decreased expression of the RIG-1 specific downstream target IRF7 enhances HIV-1 and RelA transcription in cervical tissues

**Fig 5:**
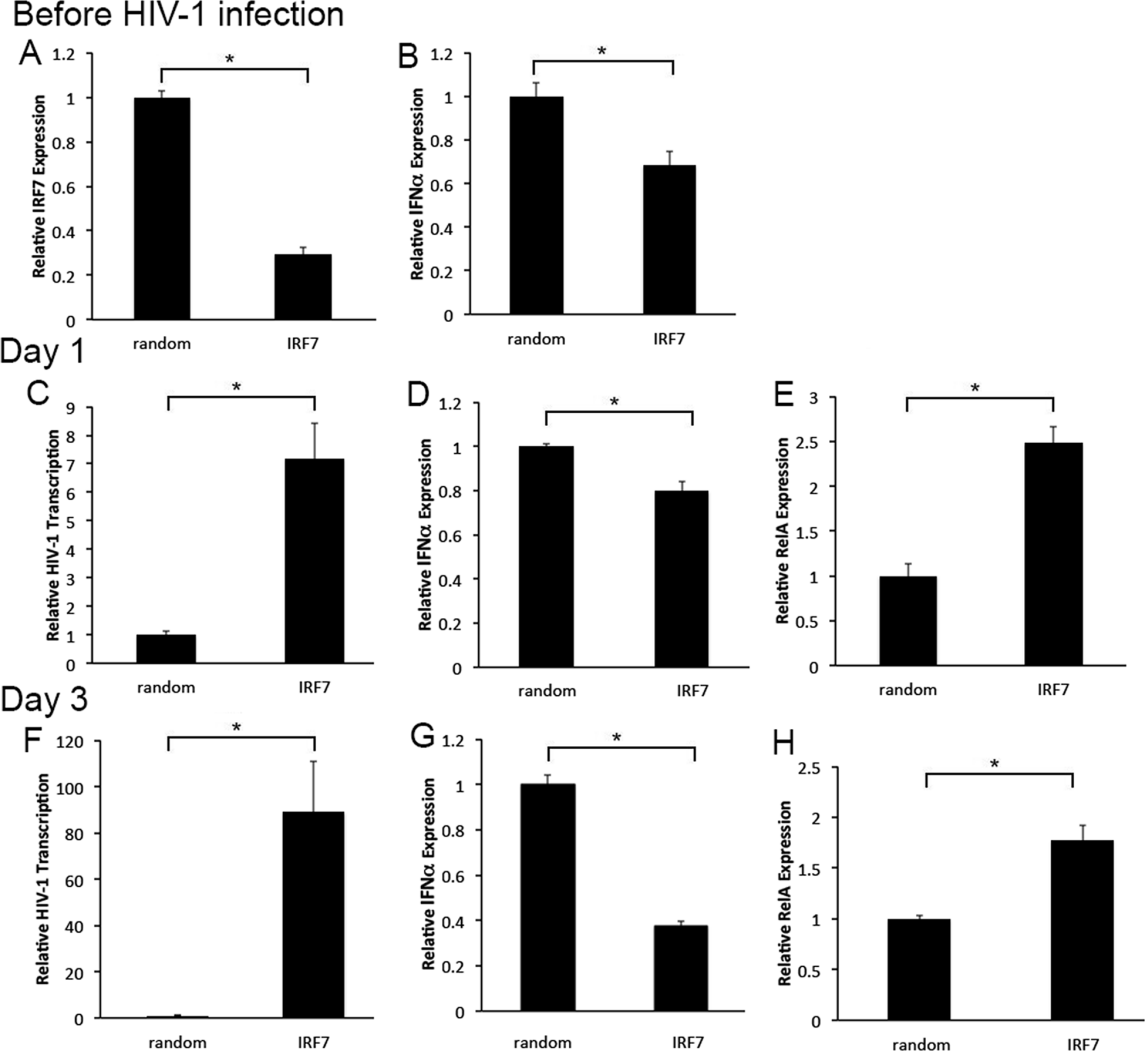
Decreasing IRF7 expression enhances HIV-1 and RelA transcription. (A) IRF7, (C and F) HIV-1, (B, D and G) IFNα and (E and H) RelA transcription in HIV-1-infected cervical tissues treated with random and IRF7 targeting siRNA were quantified by RT-PCR before (A) and (B) and on days 1 (C, D and E) and 3 (F, G and H) after HIV-1 infection. All data was normalized to GAPDH. Results were consistent among four donors and are shown as the mean ± STDEV from one representative experiment with each condition tested in triplicate. * p<0.05 for tissues treated with random and IRF7 targeting siRNA.

Our published findings demonstrate that decreasing RelA mediated pro-inflammatory responses down-regulates HIV-1 transcription in ectocervical tissues [27]. The role of IRF7 in modulating HIV-1 gene expression in cervical tissues is unknown. Given that enhanced IRF7 expression at day 5 correlated with decreased HIV-1 transcription and p24 release in poly (I:C) treated tissues and PBMCs, we evaluated the impact of down-regulating IRF7 expression on HIV-1 transcription. In these experiments, cervical tissues were treated with either random (r) or IRF7 specific siRNA for 2 days prior to overnight infection with HIV-1. Total tissue RNA was isolated before and on days 1 and 3 after infection, and evaluated for IRF7, IFNα, HIV-1 and RelA transcription. Before HIV-1 infection, we found a 60% decrease in IRF7 expression (Fig 5A), which was associated with decreased expression of the RIG-1/IRF7 specific downstream target IFNα in IRF7siRNA treated compared with rsiRNA treated tissues through day 3 (Fig 5B, 5D and 5G). Consistent with this decrease in anti-viral responses, HIV-1 transcription was enhanced by 7- and 120-fold in IRF7siRNA treated compared with rsiRNA treated tissues on days 1 and 3 after infection, respectively (Fig 5C and 5F). The increase in HIV-1 transcription was associated with enhanced RelA expression through day 3 (Fig 5E and 5H).

### Poly (I:C) improves the efficacy of TFV

Results thus far suggest that poly (I:C) down-regulated HIV-1 replication by enhancing IRF7 mediated anti-viral responses and decreasing RelA expression. Since inflammatory responses decrease the anti-HIV-1 activity of TFV at suboptimal concentrations in ectocervical tissues [25], we evaluated the potential for poly (I:C) to improve the efficacy of this microbicide. Suboptimal concentrations of TFV may be possible during periods of poor adherence, inconsistent drug use, or in the presence of additional sexually transmitted pathogens (STPs) [28,29]. In these experiments, cervical tissues were treated with poly (I:C) overnight. The next day, tissues were exposed to TFV at 10 μg/ml (suboptimal inhibitory concentrations in this system) for 6 hrs, and infected with HIV-1. After an overnight incubation, tissues were washed and cultured for 21 days. HIV-1 p24 levels were evaluated in the culture supernatants on days 0, 11 and 21 after infection. HIV-1 reverse transcription and viral integration were evaluated in genomic tissue DNA isolated on days 11 and 21. Controls included untreated tissues and those treated with TFV alone.

**Fig 6:**
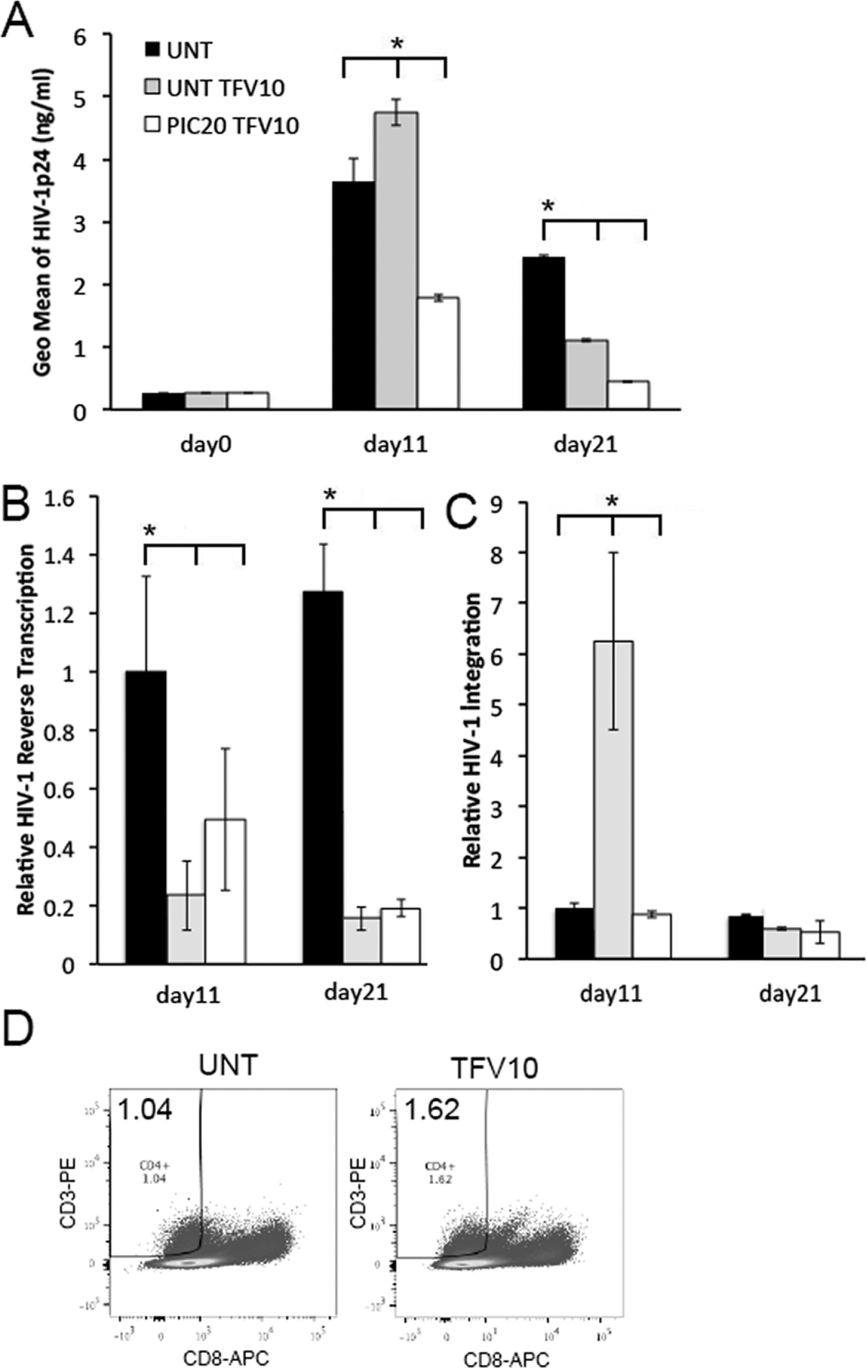
Poly (I:C) improves the efficacy of TFV in cervical tissues. (A) HIV-1 p24 levels (ng/ml) in HIV-1 infected cervical tissues left untreated or treated with TFV at 10 μg/ml alone or in combination with poly (I:C) at 20 μg/ml were evaluated after washing the residual input virus (day 0), and again on days 11 and 21 after infection. (B) Levels of HIV-1 reverse transcription and (C) viral integration in donor matched HIV-1 infected cervical tissues left untreated or treated with TFV at 10 μg/ml alone or in combination poly (I:C) at 20 μg/ml were quantified by RT-PCR on days 11 and 21 after infection. All data was normalized to human β-actin. For HIV-1 reverse transcription and integration, day 11 values in untreated control tissues were set to 1. Day 11 values in TFV or TFV/Poly (I:C) treated tissues or days 21 values in untreated; TFV or TFV/Poly (I:C) treated tissues were normalized to 1. Results were consistent among 4 donors and are shown as the mean ± STDEV from one representative experiment with each condition tested in triplicate. * p<0.05 for untreated, poly (I:C) and TFV treated tissues. (D) FACS analysis of single cell suspensions from HIV-1 infected ectocervical tissues left untreated or treated with TFV at 10 μg/ml and stained for CD3 and CD8 on day 11 after infection. CD4^+^ T cells were defined as CD3^+^ and CD8^-^. For each panel the percentage of CD4^+^ T cells from the total cell number is depicted in the upper left corner. Results were consistent among 4 donors.

On day 11, we detected transiently increased levels of HIV-1 replication in tissues treated with TFV alone compared with those in untreated control tissues (Fig 6A). Conversely, tissues treated with poly (I:C) in combination with TFV exhibited decreased HIV-1 replication compared with either untreated or TFV treated tissues (Fig 6A). On day 21, HIV-1 replication was decreased in TFV treated compared with untreated control tissues, yet the lowest levels of HIV-1 p24 release were seen in tissues co-treated with poly (I:C) and TFV.

On day 11, HIV-1 reverse transcription was decreased to comparable levels in tissues treated with TFV alone or in combination with poly (I:C) compared with those in untreated control tissues, suggesting that TFV reduced HIV-1 reverse transcription as expected (Fig 6B). This effect was sustained through day 21. At this point, tissues treated with TFV alone or in combination with poly (I:C) had decreased levels of HIV-1 reverse transcription compared with those in untreated control tissues (Fig 6B).

On day 11, we detected a 6-fold increase in HIV-1 integration in TFV treated compared with untreated control tissues. This effect disappeared by day 21, when TFV alone or in combination with poly (I:C) had the same level of HIV-1 integration than controls (Fig 6C). To evaluate whether enhanced HIV-1 integration at day 11 reflected a higher frequency of CD4^+^ T cells, we compared the percentage of CD4^+^ T cells between TFV-treated and untreated HIV-1 infected tissues by FACS. On day 11, we detected enhanced frequency of CD4^+^ T cells in TFV-treated HIV-1 infected tissues compared with untreated control tissues (Fig 6D). Thus, enhanced HIV-1 p24 levels in TFV-treated tissues on day 11 were associated with increased number of HIV-1 target/infected cells. On day 21, we detected similar levels of HIV-1 integration in all experimental conditions. TFV and TFV + poly (I:C) inhibited HIV-1 replication (p24) at day 21.

When tissues were exposed to TFV at protective concentrations ranging from 50 to 100 μg/ml in this experimental model, poly (I:C) had no additional inhibitory effect on decreasing HIV-1 replication (data not shown).

## Discussion

Mucosal inflammation increases HIV-1 replication and likely limits the anti-HIV-1 activity of microbicide candidates *in vivo* [25,30]. Suppressing inflammation in the cervicovaginal mucosa, the main portal of HIV-1 entry during heterosexual transmission has been proposed as a strategy to decrease HIV-1 acquisition and likely improve microbicide efficacy [30]. Using *ex vivo* cervical tissues, we provide novel insights on poly (I:C) regulation of HIV-1 replication at mucosal sites. Induction of IRF7 by poly (I:C) decreases RelA and HIV-1 expression. Blocking IRF7 expression by siRNA increased RelA and HIV-1 expression. Reduced HIV-1 replication likely improved the efficacy of TFV at suboptimal concentrations.

### Poly (I:C) enhances IRF7 dependent anti-viral responses

Viral recognition by epithelial and immune cells results in expression of type I IFN secondary to induction and/or nuclear translocation of IRF family members, mainly IRF3 and IRF7 [31]. We report that poly (I:C) enhanced IRF7 expression in cervical tissues. Poly (I:C) mediated increase in IRF7 expression correlated with enhanced IFNα and decreased HIV-1 transcription (Fig 2A, 2C and 2G). We also found a clear correlation between enhanced IRF7 and decreased HIV-1 expression following poly (I:C) stimulation of PBMCs (Fig 3A and 3C). We acknowledge differences in phenotype between PBMCs and cervical mucosa leukocytes [32,33]. Given that the pattern of IRF7 expression in PBMCs however reflected that detected in cervical tissues on day 5, our data underscore the role of immune cells in inducing IRF7 specific anti-viral responses in cervical tissues. This assumption is consistent with enhanced expression of the IRF7 specific target IFNα in cervical tissues (Fig 2G).

Poly (I:C) has been reported to decrease HIV-1 replication in PBMCs, imDC, MDM and human lymphoid tissue [11–14]. While decreased HIV-1 replication in imDC was due to APOBEC3G activation [12], poly (I:C) down-regulated HIV-1 replication in MDM by activating TLR3 and enhancing expression of Type I IFN and several IRFs particularly IRF7 [11]. Our findings in cervical tissues demonstrated enhanced TLR3 expression by poly (I:C) only on day 3 after infection (Fig 2F), which was associated with no change in HIV-1 RNA expression in poly (I:C) treated compared with untreated control tissues (Fig 2A). Likewise on day 3, we detected greater levels of TLR3 expression in poly (I:C) treated PBMC (Fig 3F), a time point where we saw no differences in HIV-1 transcription between poly (I:C) treated and untreated control cells (Fig 3A). Given that IRF7 is a specific downstream target of RIG-1 [4], and that TLR3 activates IRF3 and RelA rather than IRF7 [1,3,6], we postulate that poly (I:C) enhances IRF7 expression by stimulating the RIG-1/MDA5 signaling pathway. This hypothesis is consistent with detection of enhanced IRF7 and RIG-1 expression in both cervical tissues and PBMC (Fig 2C and 2E, and Fig 3C and 3E; respectively). Though IRF7 RNA expression is not a clear indication of transcription factor activation, fluctuation in IRF7 transcription was positively correlated with variations in levels of the IRF7 downstream specific target IFNα, suggesting that transcription factor expression correlated with activation (Fig 2C and 2G and Fig 5A, 5B, 5D and 5G).

IRF family members are key regulators of the immune response to viral infection [34–38]. It is unlikely that poly (I:C) may have decreased HIV-1 replication by only activating IRF7. Consistent with reports in macrophages, we saw no changes in IRF3 expression by poly (I:C) in cervical tissues (Fig 2D). Given that IRF3 is activated by phosphorylation [39–42], we cannot rule out the possibility of IRF3 activation by poly (I:C).

We also found enhanced IRF1 expression in poly (I:C) treated compared with untreated control cervical tissues (data not shown), a result that is supported by reports of increased IRF1 transcription in poly (I:C) treated MDM [11]. Thus, to address a potential redundancy among IRF family members in modulating HIV-1 expression, in a critical proof of concept experiment we found that decreasing IRF7 transcription resulted in 7- and 120-fold increase in HIV-1 transcription on days 1 and 3 after infection (Fig 5C and 5F). Furthermore, decreasing IRF7 expression resulted in down-regulation of the specific downstream target IFNα (Fig 5B, 5D and 5G). We focused on targeting IRF7 by siRNA rather than RIG-1 because RIG-1 signaling pathway also activates IRF3 and expression levels of this transcription factor were not changed by poly (I:C).

Taken together, our results underscore the impact of IRF7 specific anti-viral responses in decreasing HIV-1 replication in cervical tissues.

### Poly (I:C) decreases RelA expression

Our published results demonstrate that down-regulating RelA expression decreases HIV-1 transcription in ectocervical tissues [27]. Thus, the reduction in RelA RNA expression detected in poly (I:C) treated cervical tissues on day 5 after infection may have also contributed to reduce HIV-1 transcription (Fig 2A and 2B). Moreover, results from our siRNA experiments demonstrated that down-regulating IRF7 expression (Fig 5A) resulted in enhanced RelA transcription (Fig 5E and 5H), an outcome that was associated with an increase in HIV-1 RNA levels (Fig 5C and 5F). Thus, our data suggests that poly (I:C) stimulation of IRF7 expression is directly associated with decreased RelA expression.

Mechanisms of poly (I:C) regulation of RelA expression in PBMCs seem to be different from those in cervical tissues. We detected a poly (I:C) dependent increase in RelA, RIG-1 and TLR3 expression in PBMCs (Fig 3B, 3E and 3F), which was associated with enhanced IRF7 and IRF3 transcription (Fig 3C and 3D). This pattern of receptor and transcription factor expression correlated with decreased HIV-1 transcription (Fig 3A), suggesting that IRF7/IRF3 activated anti-viral responses counteracted the poly (I:C)–mediated enhancement in RelA expression in PBMCs. Differences in RelA expression between poly (I:C) treated tissues and PBMC, likely indicate that populations other than immune cells down-regulate RelA expression in cervical tissues on day 5 after infection. Poly (I:C) has been reported to induce RelA expression in genital epithelial cells following 30 minutes or 24 hr of treatment [43,44]. Kinetics of RelA expression at later time point were not reported in these studies. Thus, our data suggest that poly (I:C) simulation of non-immune cells decreases tissue RelA expression over time.

Evaluating gene expression mainly at the RNA level is a limitation, when protein expression was assessed for variables such as HIV-1, viral RNA expression was strongly associated with HIV-1 p24 release in both untreated and poly (I:C) treated tissues and PBMC (Figs 1A and 4). These findings support an association between RNA and protein expression in our experimental system.

### Poly (I:C) improves TFV efficacy

Poly (I:C) induced anti-viral responses and reduced RelA expression in cervical tissues prompted us to assess whether poly (I:C) could improve the efficacy of semi-protective concentrations of TFV, the leading anti-HIV microbicide. We selected partially inhibitory concentrations because they may be possible under conditions of inadequate drug use [28]. Furthermore, when poly (I:C) was used in combination with TFV at protective concentrations, we found no additional synergistic effect (data not shown).

Inflammatory responses to STPs recruit activated CD4^+^ T cells. Thus, by increasing the turnover of mucosal HIV-1 target cells, STPs may enhance HIV-1 infection and decrease the effectiveness of TFV. This hypothesis is consistent with our published findings demonstrating that inflammation triggered by HSV-2, one of the most prevalent co-infections in HIV-1 positive individuals, increases the number of activated CD4^+^ HIV-1 target cells [25]. Under settings of *ex vivo* HSV-2 co-infection, increased TFV concentrations are needed to prevent HIV-1 infection and replication compared with tissues infected with HIV-1 alone [25].

A combination of poly (I:C) and TFV demonstrated an earlier (day 11) and greater protective effect against HIV-1 compared with tissues treated with TFV alone (Fig 6A). Indeed, on day 11 TFV transiently increased HIV-1 replication possibly by enhancing the number of HIV-1 target/infected cells (Fig 6A, 6C and 6D). Although as expected these cells had low levels of reverse transcription (Fig 6B) our findings in this *ex vivo* cervical tissue explant model suggest that suboptimal concentrations of TFV do not fully prevent viral DNA production and integration in HIV-1 target cells, which in turn produced and released virions (Fig 6A). In contrast, by reducing the number of HIV-1 infected cells on day 11, poly (I:C) improved the anti-HIV-1 activity of TFV. Thus, when used in combination with partially inhibitory TFV concentrations, poly (I:C) increased anti-viral responses and decreased RelA expression diminishing the number of HIV target cells and virus release from these cells.

It is possible that other microbicide candidates or systemic pre-exposure prophylactic (PrEP) agents exert the same effect. We therefore postulate that by decreasing proliferation or activation of HIV-1 target cells, poly (I:C) will improve the anti-HIV-1 activity of topical or systemic microbicides under settings of HSV-2 and other cervicovaginal co-infections. Because of its anti-viral properties, poly (I:C) may be effective in controlling replication of additional viruses such as HSV-2. Experiments are underway to test these hypotheses.

In summary, this is the first report demonstrating that poly (I:C) increases IRF7 dependent anti-viral responses and decreases RelA expression in cervical tissues. Enhanced anti-viral responses and IRF7 down-regulation of RelA expression likely results in decreased HIV-1 replication and improved TFV’s efficacy specifically early during infection and in the presence of suboptimal TFV concentrations.

Understanding the interaction between pro-inflammatory and anti-viral responses in the genital mucosa will provide crucial insights for the identification of targets that can be harnessed to protect from sexually transmitted pathogens in general and viral infections such as HIV-1 and HSV-2 in particular. Combining immune modulators with antiretrovirals or other antiviral compounds may be a feasible strategy to improve topical and systemic PrEP efficacy.
