# Correction: Enhancing Interferon Regulatory Factor 7 Mediated Antiviral Responses and Decreasing Nuclear Factor Kappa B Expression Limit HIV-1 Replication in Cervical Tissues

**DOI:** 10.1371/journal.pone.0133815

**Published:** 2015-07-20

**Authors:** The PLOS ONE Staff

The first author’s name is spelled incorrectly. The correct name is Christiane Rollenhagen. The correct citation is: Rollenhagen C, Macura SL, Lathrop MJ, Mackenzie TA, Doncel GF, Asin SN (2015) Enhancing Interferon Regulatory Factor 7 Mediated Antiviral Responses and Decreasing Nuclear Factor Kappa B Expression Limit HIV-1 Replication in Cervical Tissues. PLoS ONE 10(6): e0131919. doi:10.1371/journal.pone.0131919

## References

[1] Rollenhage C, Macura SL, Lathrop MJ, Mackenzie TA, Doncel GF, Asin SN (2015) Enhancing Interferon Regulatory Factor 7 Mediated Antiviral Responses and Decreasing Nuclear Factor Kappa B Expression Limit HIV-1 Replication in Cervical Tissues. PLoS ONE 10(6): e0131919 doi:10.1371/journal.pone.0131919 26121689 10.1371/journal.pone.0131919 PMC4485897

